# Mental Health in Young Adult University Students During COVID-19 Lockdown: Associations with Physical Activity, Sedentary Behaviors and Sleep Quality

**DOI:** 10.3390/ijerph22020241

**Published:** 2025-02-08

**Authors:** Gustavo Silva, Francisco Estima, Ana Carvalhinho Silva, Jorge Mota, Clarice Martins, Luísa Aires

**Affiliations:** 1 Research Centre in Sports Sciences, Health Sciences and Human Development (CIDESD), University of Maia (UMaia), 4475-690 Maia, Portugal; festima.dcd@umaia.pt (F.E.); acarvalhinho@umaia.pt (A.C.S.); 2 Research Centre in Physical Activity, Health and Leisure (CIAFEL), Faculty of Sport, University of Porto (FADE-UP), 4050-313 Porto, Portugal; jmota@fade.up.pt (J.M.); clarice@fade.up.pt (C.M.); luisaaires.7566@esag-edu.net (L.A.); 3 Laboratory for Integrative and Translational Research in Population Health (ITR), 4050-091 Porto, Portugal

**Keywords:** confinement, social distance, screen time, sitting time, social networks, social media, moderate physical activity, vigorous physical activity, 24 h movement behaviors

## Abstract

This study analyzed associations between physical activity (PA), sedentary behaviors (SB), sleep, and mental health indicators in young adults during the COVID-19 lockdown (15 January–19 April 2021). The participants were 549 Portuguese university students (18–30 years, 57.7% male). Mental health was assessed using the DASS-21 for depression, anxiety, stress, and DASS-21 total score (DASSt). Physical activity (vigorous, moderate, walking) was measured with the IPAQ, while SB (e.g., sitting, screen time) and sleep were evaluated using self-reported measures and the Pittsburgh Sleep Quality Index (PSQI). Multiple linear regressions showed that sleep quality (β = 2.116), sitting time (ST; β = 0.451), vigorous PA (VPA; β = −0.005), and computer use for work/study (β = 0.444) were significantly associated with overall mental health, DASSt (*R*^2^ = 0.358). Sleep quality, sitting, and VPA were also linked to depression (*R*^2^ = 0.308), anxiety (*R*^2^ = 0.275), and stress (*R*^2^ = 0.338). Computer use for work/study was additionally associated with anxiety and stress. Overall, poor sleep quality, increased sitting, and computer use for work/study, alongside lower VPA, showed significant associations with poorer mental health outcomes. These findings highlight the importance of sleep, PA, and limiting sedentary behaviors, particularly during lockdowns, in mitigating mental health issues among Portuguese young adults.

## 1. Introduction

With the emergence of the first cases of the Coronavirus Disease (COVID-19) in China, several countries have implemented preventive measures, including restrictions on people’s mobility and travel, social distancing, lockdown periods, quarantines, and prophylactic isolation, following recommendations from the World Health Organization (WHO) [1].

In Portugal, March 2021 marked the third wave of COVID-19 transmission, accompanied by a second prolonged lockdown. This period included the suspension of numerous economic sectors, businesses, and services, as well as the closure of gyms, sports clubs, and public spaces with high attendance. In the education sector, for example, primary and secondary schools, early childhood education, and higher education institutions transitioned to distance and remote learning as of 22 January 2021 [2]. These new regulations, which reshaped daily life both in Portugal and globally, significantly altered personal, social, and professional routines, profoundly affecting lifestyle habits and behaviors, with substantial implications for physical and mental health [3].

One major consequence of the COVID-19 pandemic has been global psychological distress. In this regard, some researchers have reported an increase in the prevalence of pandemic-related psychiatric morbidity and psychological suffering [4,5]. During this period, heightened rates of anxiety and stress-related disorders were often associated with the pandemic itself, such as fear of personal or familial infection and generalized uncertainty about the future [6]. Evidence also suggests that quarantine or prophylactic isolation contributed to the deterioration of mental health [4].

Developing healthy coping and resilience strategies during the pandemic was a significant challenge. However, exploring approaches to improve psychological well-being during the restrictions imposed by the pandemic was both necessary and challenging [7].

One potential coping strategy during pandemics and mandatory lockdowns is physical activity. The benefits of physical activity for overall health, including physical, bone, muscular, cardiovascular, and mental health, are well documented in the evidence underpinning the WHO’s international recommendations on this matter [8]. The evidence highlights the importance of encouraging populations to increase their levels of physical activity and reduce sedentary behaviors, as sedentary lifestyles can be harmful even when individuals meet the recommendations for moderate-to-vigorous physical activity [9,10]. Large population studies report that individuals who engage in higher levels of physical activity typically experience fewer mental health problems in the preceding month compared to their less active peers. This association between physical activity and mental health indicators is independent of age, sex, race, income, socioeconomic status, and type of physical activity performed [11].

Several studies have examined how the COVID-19 pandemic has affected physical activity and the subsequent implications for mental health. Evidence suggests a reduction of approximately 33% in habitual physical activity during the early stages of the pandemic, between February and April 2020, in the general adult population [12]. The restrictive measures imposed during the pandemic limited outdoor physical activities, forcing individuals to exercise at home, which largely explains the significant decrease in physical activity levels during this period [13,14].

Focusing on young adults, evidence shows that university students were more sedentary, anxious, and depressed during the winter break of 2020, with sedentary behavior increasing during the second week of this period [15]. Another study involving university students suggested that strategies to promote mental health during the COVID-19 pandemic should include minimum daily physical activity levels and healthy sleep hygiene habits to enhance mental health [16]. However, some studies have reported contradictory findings. For instance, in one study involving university students, physical activity largely decreased, but a significant portion of the sample managed to maintain or even increase their physical exercise [17]. Additionally, in another study, physical activity decreased during the first week of lockdown but gradually increased as individuals adapted their habits and lifestyles to the restrictive measures and distancing protocols [18]. With that said, it appears logical to invest in gathering additional evidence to clarify the relationships between physical activity, sedentary behaviors, sleep quality, and mental health during the pandemic period, particularly during phases of lockdown and stricter restrictive measures. Such evidence would be relevant for determining strategies to address potential future lockdowns and social distancing measures during future pandemics, with the goal of promoting mental health through recommendations focused on adequate sleep habits and a more active, less sedentary lifestyle.

Therefore, the main purpose of this study was to analyze the associations between habitual physical activity, sedentary behaviors, sleep quality, and mental health indicators during a lockdown period in the COVID-19 pandemic among young adult university students.

## 2. Materials and Methods

### 2.1. Subjects

This study was developed in accordance with the Portuguese General Data Protection Regulation (GDPR) and approved by the Ethics Committee of the University of Maia on 21 April 2021 (registration number 38/2021). This research project, designed as a cross-sectional observational study, involved a voluntary sample of 586 participants aged between 18 and 55 years. The sample consisted of higher education students from public or private universities and polytechnic institutions within the Portuguese national territory who were at least 18 years old and regularly enrolled in one of the three levels of study cycles: undergraduate (1st cycle), master’s (2nd cycle), and doctoral (3rd cycle) programs. The study was conducted in compliance with the Declaration of Helsinki and followed ethical principles of research involving human participants. All participants received a written explanation of the project’s objectives, procedures, risks, and benefits, and their anonymity and data confidentiality were guaranteed. All participants agreed to the terms of informed consent, confirming their voluntary participation and authorizing the use of their data for scientific research purposes.

All procedures for completing informed consent forms and collecting data through questionnaires were carried out digitally online using the Google Forms platform. Access to volunteers was primarily achieved through their higher education institutions (HEIs), using institutional email addresses and announcements on the institutions’ websites and social media platforms. Initially, the responsible researchers contacted the management bodies of the HEIs, requesting authorization and support for disseminating the project. Once authorization was obtained from each HEI, a standard email was sent to the academic community of the respective institution, including information about the project and a link to access the informed consent forms and corresponding questionnaires.

All data collection was conducted using the Google Forms platform between 22 April and 25 June 2021. The data were collected retrospectively, recalling and referencing the second confinement period (lockdown) defined by Portuguese authorities, which lasted from 15 January 15 to 15 March 2021 (extended to 19 April for higher education students).

For the current study, data were selected from participants aged 18 to 30 years, resulting in a final sample of 549 individuals, of whom 232 were female (42.3%) and 317 were male (57.7%).

### 2.2. Variables and Instruments

Height and body mass were self-reported in centimeters and kilograms. The Body Mass Index (BMI) was calculated as the ratio of body mass (kg) to the square of height (m^2^).

To measure indicators of mental health, the Depression Anxiety Stress Scale (DASS-21) was used in the Portuguese version [19,20]. The DASS-21 consists of a questionnaire made up of 21 items organized into three sub-scales: depression (DEP), anxiety (ANX), and stress (STR). Each scale consists of 7 items. Each item expresses the extent to which participants recently experienced each mental health symptom using a 4-point Likert scale of severity or frequency: 0 = did not apply to me at all; 1 = applied to me some of the time; 2 = applied to me most of the time; and 3 = applied to me nearly all the time. The DASS-21 total score (DASSt) was calculated by the sum of the three sub-scales. Each sub-scale score (DASSt, ANX and STR) was later classified, according to symptoms severity, into normal, mild, moderate, severe and extremely severe to characterize participants’ mental health status during this lockdown period following DASS-21 scoring recommendations [21]. For the DASS Total Score (DASSt), as well as the depression (DEP), anxiety (ANX), and stress (STR) subscales, lower scores indicate better mental health outcomes, whereas higher scores reflect poorer mental health outcomes.

Sedentary behaviors were measured through a set of questions about sitting time and exposure to screen-based electronic devices, such as TV, personal computers, tablets, and smartphones, during weekdays and weekends [22,23]. Sitting time (ST), time spent on TV (TVt), time spent on personal computers time for work/study (PCWSt), time spent on personal computers time for leisure (PCLt), time spent on tablets (TABt), and time spent on smartphone (SPHt) were all evaluated. The Total Screen Time (STt) was calculated from the sum of TVt, PCWSt, PCLt, TABt, and SPHt. Additionally, time spent on social networks (SNt) was also assessed as an additional indicator of sedentary behaviors during the confinement period. The average daily time (hours per day, h·day^−1^) was calculated, and a maximal value of 960 min per day (16 h·day^−1^) was assumed for each sedentary behavior.

Physical activity (PA) was measured using the International Physical Activity Questionnaire (IPAQ) in its shortened form [24,25]. Through the IPAQ, an average daily time (min·day^−1^) and the weekly time (min·week^−1^) were estimated for vigorous physical activity (VPA), moderate physical activity (MPA), and walking time (WPA), which, when combined, represent total physical activity as an indicator of MVPA (moderate to vigorous physical activity). Additionally, following the international recommendations for physical activity [26], participants were classified as “inactive” if they did not meet the 150 min·week^−1^ of MVPA and “active” if they engaged in 150 min·week^−1^ or more. For each PA intensity, a maximal of 180 min·day^−1^ was assumed.

To measure the duration and quality of sleep, the Pittsburgh Sleep Quality Index (PSQI) was used [27,28,29]. The PSQI questionnaire consists of a set of 19 items/questions organized to produce scores for 7 components: (1) Subjective Sleep Quality; (2) Sleep Latency; (3) Sleep Duration; (4) Habitual Sleep Efficiency; (5) Sleep Disturbances; (6) Use of Sleep Medication; and (7) Daytime Dysfunction. For each of these 7 components, scores are expressed on a scale from 0 to 3, where the lowest value (0) represents “superior” quality or efficiency and the highest value (3) represents “inferior” quality or efficiency. The sum of the scores in the 7 components allows for the calculation of the total PSQI score, with a range from 0 to 21. In the total PSQI, lower values reflect better sleep quality, while higher values denote poorer sleep quality. A total PSQI score of 5 or below is indicative of “good sleep quality”, whereas a score greater than 5 signifies “poor sleep quality”.

### 2.3. Statistics

Descriptive data for continuous variables are presented as mean and standard deviation (Mean ± SD). An Independent Students’ *t* test was used to analyze differences between sex. Pearson correlations and partial correlation analyses were used to explore the relationships between depression, anxiety, stress, and DASSt scales with each indicator of sedentary behaviors, physical activity, and sleep quality. Partial correlation analyses were carried out, being adjusted by age and sex as confounders. Multiple linear regression analyses were carried out with Enter and Stepwise methods to test if physical activity, sedentary behaviors, and sleep combined were associated with symptoms of depression, anxiety, stress and DASSt. Results for multiple linear regressions (MLRs) are presented as unstandardized (beta, β) coefficients and respective 95% confidence intervals for each independent variable (predictors). The *R* squared (*R*^2^, variance explained) is reported for each regression model as an overall indicator of model fit. Data are presented prioritizing results from MLRs with the Stepwise method to identify which independent variables were strongly related with mental health outcomes. All analyses were completed with SPSS version 29, with a significance level of 0.05 (*p* ≤ 0.05).

## 3. Results

### 3.1. Participants’ Descriptive Characteristics

The current analysis included 549 university students aged 18–30 years. The sample comprised 57.7% men, with 81.4% undergraduate students, 17.5% master’s students, and 1.1% doctoral candidates. Approximately 73% were enrolled in Sports Sciences programs, 8.8% were enrolled in Psychology and Behavioral Sciences, and 20.5% in other academic fields.

Overall, 84.2% of participants had a normal weight, 14.2% were classified as overweight, and 1.6% as obese. The majority (91%) were physically active, reporting at least 150 min of moderate-to-vigorous physical activity (MVPA) per week. However, 53% were categorized as having poor sleep quality, and 56.3% reported sleeping less than 8 h per day. Regarding mental health markers, 40.6% exhibited symptoms of depression (15.1% mild, 14.2% moderate, 4.9% severe, and 6.4% extreme), 29.9% showed symptoms of anxiety (11.3% mild, 7.5% moderate, 4.2% severe, and 6.9% extreme), and 27.5% reported symptoms of stress (10.6% mild, 6.2% moderate, 7.3% severe, and 3.5% extreme).

Table 1 presents participants’ characteristics and descriptive statistics for the total sample and by sex. Significant differences (*p* ≤ 0.05) were observed between men and women across several variables. Men were younger and exhibited higher values for height, weight, and BMI. Men also reported spending more time on personal computers use for leisure, vigorous physical activity (VPA), walking, and total moderate-to-vigorous physical activity (MVPA). Conversely, mental health indicators were generally poorer among women, who reported higher scores for depression, anxiety, stress, and the DASS-21 total score. Women also spent more time on personal computers for work and study, smartphones, and social networks, and they also had poorer sleep quality, as indicated by higher PSQI scores.

### 3.2. Correlations Between Sedentary Behaviors, Physical Activity, Sleep Quality and Indicators of Depression, Anxiety and Stress

Table 2 presents Pearson’s correlations and partial correlations (adjusted for age and sex) describing the associations between mental health indicators—depression, anxiety, stress, and DASS-21 total score—and sedentary behaviors, physical activity, and sleep quality. Depression, anxiety, stress, and DASS-21 scores were positively correlated (*p* ≤ 0.05) with PCWSt, SPHt, STt, and sitting time, while SNt was positively correlated (*p* ≤ 0.05) only with anxiety and the DASS-21 total score. Vigorous PA, MPA, and MVPA were inversely correlated (*p* ≤ 0.05) with depression, anxiety, stress, and the DASS-21 total score. Walking was inversely correlated (*p* ≤ 0.05) only with depression. Sleep quality (PSQI), was positively correlated (*p* ≤ 0.05) with depression, anxiety, stress, and the DASS-21 total score. After adjusting for age and sex, these correlations remained significant (*p* ≤ 0.05) for the following: depression with SPHt, STt, sitting, VIGPA, MPA, MVPA, and PSQI; anxiety with PCWSt, SPHt, STt, sitting, VIGPA, MVPA, and PSQI; stress with PCWSt, SPHt, STt, SNt, sitting, VIGPA, and PSQI; and the DASS-21 total score with PCWSt, SPHt, STt, sitting, VIGPA, MPA, MVPA, and PSQI. Overall, these findings suggest that increased time spent engaged in sedentary behaviors is associated with higher (poorer) levels of depression, anxiety, stress, and overall mental health. In contrast, greater engagement in VIGPA, MPA, and MVPA is associated with lower (better) levels of depression, anxiety, stress, and overall mental health.

### 3.3. Linear Regression Models Describing Multiple Relationships Between Sedentary Behaviors, Physical Activity, Sleep Quality, and Indicators of Depression, Anxiety and Stress

Table 3 presents the results of multiple linear regression analyses conducted using the Stepwise method. In the first set of analyses, depression, anxiety, stress, and the total DASS-21 score were treated as dependent variables (outcomes), while sedentary behaviors, physical activity, and sleep quality were included as independent variables (predictors). In the second set of analyses, age and sex were added as independent variables (covariates). For each regression analysis, only the final step, representing the best-fitting model, is reported. Even when age and sex were forced into the regression models as covariates, only sex remained a significant predictor of mental health indicators in the final models, alongside physical activity, sedentary behaviors, and sleep quality.

Prior to adjustments for age and sex, sleep quality (PSQI), sitting time, and VPA were significantly associated with depression. Sleep quality, sitting time, VPA, and PCWSt were associated with anxiety, while PSQI, PCWSt, and VPA were associated with stress. Similarly, PSQI, sitting time, VPA, and PCWSt were associated with DASSt. After including age and sex as covariates in the Stepwise regression models, PSQI, sitting time, VPA, and sex were significantly associated with depression. Sleep quality, sex, sitting time, and VPA were associated with anxiety, while PSQI, sex, PCWSt, and sitting time were associated with stress. For the DASSt, PSQI, sex, sitting time, and VPA remained significant predictors.

## 4. Discussion

The results show that sleep quality, sedentary behaviors such as sitting time, and time spent on PC working or studying are significantly associated with depression, anxiety, and stress in Portuguese young adults during lockdown. Poor sleep quality, measured by the total PSQI, showed a higher association with poorer levels of mental health, as vigorous PA showed a protective effect, evidenced by its inverse association with mental health indicators.

Before the pandemic, studies indicated that the guidelines for physical activity, sedentary behavior, and sleep were associated with better mental health indicators but also identified that participants faced difficulties in achieving these recommendations [11,30].

During the COVID-19 pandemic, lockdown measures, though essential to contain the spread of the virus, profoundly changed 24 h movement behaviors, affecting both physical and mental health. These changes further accentuated existing gaps in meeting movement behavior recommendations. Studies that focused on 24 h-movement behaviors during this restrictive period reported reductions in physical activity levels, increased sedentary behaviors, and deterioration in sleep quality, which were associated with poorer mental health indicators such as anxiety, depression, and stress [14,31,32,33]. The present study corroborates this evidence, showing that behaviors such as longer sitting time and prolonged use of the PC for work/study, combined with poor sleep quality, were predictors of higher levels of depression, anxiety, and stress in young adults in Portugal.

However, the impact of the lockdown was not uniform. Factors such as gender, age, socioeconomic status, and previous levels of physical activity significantly influenced changes in 24 h behaviors and mental health, according to other studies [14,31,32]. In a study carried out in Belgium, it was concluded that, in general, there was an increase in physical activity, especially among those who were less active before confinement, but it was not reported among individuals with low education, women, and those who exercised before the pandemic with friends or in clubs [14]. Other studies have concluded that physical activity levels have decreased during confinement, while sedentary behaviors have increased. Changes in sedentary behaviors were more pronounced in men than in women [32,34]. Even studies reporting an increase in physical activity levels also reported more sitting time [14].

Poor sleep quality also emerged as an important factor associated with poorer levels of mental health, which aligns with the literature reporting similar findings on this matter [35,36]. These results highlight the importance of sleep in several human body systems and functions, including emotional regulation and stress management. Good sleep quality and an adequate number of hours of sleep are essential for regulating the autonomic nervous system, which impacts physiological and psychological balance. Promoting sleep hygiene with regular sleep schedules and reducing screen exposure before bedtime are actions that can improve sleep [35,37,38,39].

Vigorous physical activity, in this study, showed a protective effect, evidenced by its inverse association with all the mental health indicators. The literature reports that, during the lockdown, those increasing their levels of physical activity were not only able to meet the guidelines for physical activity levels but also reported a better perception of health and well-being, with fewer negative psychological symptoms and lower levels of stress [14]. However, specific types of physical activity, durations, and frequencies may be more effective than others in reducing mental health problems. In this study, vigorous intensities showed a positive association with mental health, while other studies showed that vigorous intensity, practiced at a competitive level, did not produce the same positive effect, with walking and more leisurely physical activities showing better outcomes for mental health [11,40].

With the end of the lockdown, there has been a gradual recovery in physical activity levels, particularly among men who were physically active before the pandemic, as they returned to their pre-pandemic lifestyle habits. However, women reported a decrease in physical activity levels compared to the lockdown period, during which they had reported an increase due to having more spare time at home [14,33]. Unfortunately, these patterns underscore the persistent gender inequality in physical activity levels, a disparity that has endured since before the COVID-19 pandemic [41].

These results align with the Sustainable Development Goals (SDGs) established by the United Nations, particularly SDG 3 (Good Health and Well-being), which aims to improve health and well-being through sustainable lifestyle changes. Encouraging sustainable behavioral changes and raising awareness about the full benefits of an active and balanced lifestyle are important for long-term committed people to improve their mental health and overall well-being. Thus, further studies should focus on exploring the recovery of pre-pandemic lifestyle habits and assessing which behaviors adopted during the lockdown have been maintained or reversed in the post-pandemic period. Such research will contribute to achieving SDG 3.4, which aims to reduce premature mortality due to non-communicable diseases through prevention and treatment. Also, investigating these behavioral changes will support the promotion and adoption of sustainable health behaviors and enhance inclusivity in addressing health disparities across different socioeconomic and demographic groups (SDG 10—reduced inequality).

These future perspectives are important for addressing the lasting effects of the pandemic on lifestyle habits and mental health. Suggested approaches include promoting the recovery of healthy behaviors and supporting the maintenance of the positive changes made during the lockdown. These efforts are essential for fostering resilience and improving overall well-being in the long term.

The findings of this study should be interpreted with caution. While the use of questionnaires enabled efficient large-scale data collection within a short timeframe—an invaluable advantage given the critical nature of the study period—this approach also has inherent limitations. Unlike objective measures of physical activity and 24 h movement behaviors, such as accelerometers, questionnaires are subject to potential biases due to their reliance on participants’ self-reported perceptions. This subjectivity may introduce variability and affect the accuracy of the data. Nonetheless, questionnaires offered the advantage of providing detailed insights into movement behaviors across various contexts, types, and organizational structures, which may not be as easily captured through objective measures. Ideally, combining questionnaires with objective measures, such as accelerometers, would enhance the characterization of 24 h movement behaviors and offer more robust insights, where feasible.

It is important to note that this study was conducted with a unique sample, the majority of whom (73%) were enrolled in Sports Sciences higher education programs. Additionally, 91% of participants reported being physically active during the lockdown period. As such, these findings should be interpreted within the context of a predominantly physically active population of young adult university students.

Raising awareness regarding the importance of promoting an active lifestyle, managing sedentary behaviors, and implementing healthy sleep routines is essential to mitigate negative impacts on mental health. The lockdown period has underscored the complex relationship between 24 h movement behaviors and mental health, highlighting the need to support people in achieving balance in lifestyle behaviors.

## 5. Conclusions

This study highlights the complex relationships between physical activity, sedentary behaviors, sleep quality, and mental health during the COVID-19 lockdown among young adult university students. The findings reveal that higher levels of vigorous physical activity, moderate physical activity, and moderate-to-vigorous physical activity were associated with better mental health, including lower levels of depression, anxiety, and stress. Conversely, increased sedentary behaviors, particularly sitting time, the use of personal computers for work and study, and poor sleep quality were linked to poorer mental health outcomes. Notably, women exhibited poorer mental health indicators and sleep quality compared to men. These results underscore the importance of promoting physical activity, reducing sedentary time, and improving sleep quality as critical strategies for fostering mental health in young adults, especially during challenging periods like lockdowns.

## Figures and Tables

**Table 1:** Participants’ characteristics and descriptive statistics.

	All (*n* = 549)	Male (*n* = 317)	Female (*n* = 232)	*t* Test	*p*-Value	Cohen’s d
Age (years)	20.4 ± 2.4	20.0 ± 2.2	20.9 ± 2.5	−4.570	*p* < 0.001 **	0.395
Height (cm)	172.1 ± 9.1	177.9 ± 5.9	164.2 ± 6.4	26.134	*p* < 0.001 **	−2.258
Weight (kg)	67.3 ± 11.2	72.7 ± 8.9	59.9 ± 9.7	16.174	*p* < 0.001 **	−1.397
Body Mass Index (kg·m^−2^)	22.6 ± 2.8	23.0 ± 2.5	22.2 ± 3.2	3.071	0.002 *	−0.276
DEP (score)	4.7 ± 4.7	3.9 ± 4.2	5.9 ± 5.0	−4.832	*p* < 0.001 **	0.429
ANX (score)	3.0 ± 3.5	1.9 ± 2.5	4.4 ± 4.2	−7.884	*p* < 0.001 **	0.733
STR (score)	5.4 ± 4.7	3.8 ± 3.7	7.6 ± 5.0	−9.686	*p* < 0.001 **	0.875
DASSt (score)	13.1 ± 12.0	9.6 ± 9.6	17.8 ± 13.2	−8.014	*p* < 0.001 **	0.726
TVt (h·day^−1^)	1.8 ± 1.5	1.8 ± 1.5	1.8 ± 1.4	−0.194	0.846	0.017
PCWSt (h·day^−1^)	3.9 ± 2.5	3.4 ± 2.3	4.7 ± 2.6	−6.390	*p* < 0.001 **	0.552
PCLt (h·day^−1^)	2.0 ± 2.0	2.4 ± 2.2	1.5 ± 1.6	5.813	*p* < 0.001 **	−0.478
TABt (h·day^−1^)	0.4 ± 1.0	0.3 ± 1.0	0.5 ± 0.9	−1.472	0.142	0.127
SPHt (h·day^−1^)	3.8 ± 2.8	3.6 ± 2.6	4.0 ± 3.0	−1.606	0.109	0.139
STt (h·day^−1^)	12.0 ± 5.1	11.6 ± 5.2	12.5 ± 5.0	−2.128	0.034 *	0.184
SNt (h·day^−1^)	3.0 ± 2.2	2.8 ± 2.0	3.2 ± 2.3	−2.298	0.022 *	0.199
Sitting (h·day^−1^)	6.7 ± 2.6	6.6 ± 2.6	6.8 ± 2.7	−1.099	0.272	0.095
VPA (min·week^−1^)	279.2 ± 280.6	323.0 ± 272.6	219.4 ± 280.9	4.343	*p* < 0.001 **	−0.375
MPA (min·week^−1^)	254.9 ± 278.0	268.3 ± 287.2	236.5 ± 264.3	1.325	0.186	−0.114
WPA (min·week^−1^)	279.6 ± 311.8	302.3 ± 333.2	248.7 ± 277.7	2.049	0.041 *	−0.172
MVPA (min·week^−1^)	813.7 ± 630.3	893.6 ± 645.4	704.6 ± 593.3	3.505	*p* < 0.001 **	−0.303
PSQI (score)	6.1 ± 3.0	5.5 ± 2.7	6.9 ± 3.1	−5.298	*p* < 0.001 **	0.468

Notes: DEP = depression; ANX = anxiety; STR = stress; DASSt = DASS-21 total score; TVt = TV time; PCWSt = PC for work/study; PCLt = PC for leisure; TABt = Tablet; SPHt = smartphone; SNt = social networks; STt = Total Screen Time; VPA = vigorous physical activity; MPA = moderate physical sctivity; WPA = Walking Physical Activity; MVPA = moderate-to-vigorous physical activity; PSQI = Pittsburgh Sleep Quality Index; * *p* ≤ 0.05; ** *p* ≤ 0.001.

**Table 2:** Pearson and partial correlations between sedentary behaviors, physical activity, sleep quality, and indicators of depression, anxiety, and stress.

	DEP		ANX		STR		DASSt	
	*r*	*r*_partial_	*r*	*r*_partial_	*r*	*r*_partial_	*r*	*r*_partial_
TVt	−0.033	−0.034	−0.017	−0.019	−0.013	−0.018	−0.023	−0.026
PCWSt	0.121 **	0.076	0.192 **	0.121 *	0.262 **	0.179 **	0.207 **	0.135 *
PCLt	0.035	0.085	−0.018	0.064	−0.038	0.060	−0.007	0.076
TABt	−0.046	−0.061	−0.037	−0.063	−0.060	−0.093 *	−0.053	−0.079
SPHt	0.124 **	0.108 *	0.132 **	0.109 *	0.114 **	0.097 *	0.132 **	0.113 *
STt	0.122 **	0.105 *	0.147 **	0.123 *	0.160 **	0.136 *	0.154 **	0.131 *
SNt	0.079	0.056	0.092 *	0.058	0.078	0.044	0.089 *	0.057
Sitting	0.187 **	0.181 **	0.195 **	0.191 **	0.197 **	0.194 **	0.208 **	0.204 **
VPA	−0.184 **	−0.159 **	−0.190 **	−0.147 **	−0.165 **	−0.102 *	−0.193 **	−0.147 **
MPA	−0.135 **	−0.127 *	−0.093 *	−0.079	−0.081	−0.064	−0.112 **	−0.099 *
WPA	−0.086 *	−0.068	−0.047	−0.017	−0.046	−0.014	−0.065	−0.038
MVPA	−0.184 **	−0.160 **	−0.149 **	−0.108 *	−0.132 **	−0.080	−0.168 **	−0.128 *
PSQI	0.532 **	0.508 **	0.487 **	0.445 **	0.544 **	0.511 **	0.566 **	0.533 **

Notes: DEP = depression; ANX = anxiety; STR = stress; DASSt = DASS-21 total score; TVt = TV Time; PCWSt = PC for work/study; PCLt = PC for Leisure; TABt = Tablet; SPHt = smartphone; SNt = social networks; STt = Total Screen Time; VPA = vigorous physical activity; MPA = moderate physical activity; WPA = Walking Physical Activity; MVPA = moderate-to-vigorous physical activity; PSQI = Pittsburgh Sleep Quality Index; *r* = Pearson’s correlation coefficient; *r*_partial_ = partial correlation coefficients with adjustments for age and sex as covariates; * *p* ≤ 0.05; ** *p* ≤ 0.001.

**Table 3:** Multiple linear regression for relationships between sedentary behaviors, physical activity, sleep quality, and indicators of mental health.

Dependent Variables	Without Adjustment for Age and Sex				With Adjustments for Age and Sex			
	Step	Independent Variables	β (95%CI, LB; UB)	*R* ^2^	Step	Independent Variables	β (95%CI, LB; UB)	*R* ^2^
DEP	3	(Constant)	−0.899 (−2.046; 0.248)	0.308 *	4	(Constant)	−0.390 (−1.636; 0.856)	0.313 *
		PSQI	0.791 (0.678; 0.903) **			PSQI	0.767 (0.652; 0.881) **	
		Sitting	0.198 (0.073; 0.324) *			Sitting	0.197 (0.072; 0.322) *	
		VPA	−0.002 (−0.003, −0.001) *			VIGPA	−0.002 (−0.003; −0.001) *	
						Sex	−0.706 (−1.391; −0.02) *	
ANX	4	(Constant)	−1.242 (−2.145; −0.338)	0.275 *	4	(Constant)	0.133 (−0.805; 1.071) **	0.316 *
		PSQI	0.529 (0.442; 0.616) **			PSQI	0.483 (0.397; 0.569) **	
		Sitting	0.132 (0.030; 0.234) *			Sex	−1.621 (−2.137; −1.105) **	
		VPA	−0.001 (−0.002; −0.001) *			Sitting	0.166 (0.072; 0.26) **	
		PCWSt	0.124 (0.018; 0.231) *			VIGPA	−0.001 (−0.002; −0.001) *	
STR	3	(Constant)	−0.536 (−1.493; 0.422)	0.338 *	4	(Constant)	0.579 (−0.594; 1.752)	0.398 *
		PSQI	0.807 (0.697; 0.918) **			PSQI	0.729 (0.622; 0.837) **	
		PCWSt	0.347 (0.217; 0.476) **			Sex	−2.508 (−3.168; −1.848) **	
		VPA	−0.001 (−0.003; −0.001) *			PCWSt	0.189 (0.055; 0.323) *	
						Sitting	0.158 (0.033; 0.283) *	
DASSt	4	(Constant)	−3.315 (−6.202; −0.428)	0.358 *	4	(Constant)	1.021 (−1.987; 4.029)	0.390 *
		PSQI	2.116 (1.838; 2.394) **			PSQI	1.979 (1.703; 2.255) **	
		Sitting	0.451 (0.125; 0.778) *			Sex	−4.997 (−6.651; −3.343) **	
		VPA	−0.005 (−0.008; −0.002) *			Sitting	0.575 (0.272; 0.877) **	
		PCWSt	0.444 (0.103; 0.784) *			VIGPA	−0.004 (−0.006; −0.001) *	

Notes: Regression loads for independent variables presented as unstandardized beta (β) coefficients and respective 95% confidence interval (LB = lower bound; UB = upper bound); DEP = depression; ANX = anxiety; STR = stress; DASSt = DASS-21 total score; PCWSt = PC for work/study; VPA = vigorous physical activity; PSQI = Pittsburgh Sleep Quality Index; sex (0 = female; 1 = male); * *p* ≤ 0.05; ** *p* ≤ 0.001.

## Data Availability

The data included in the present study are available upon request from the corresponding author.

## References

[1] World Health Organization Considerations in Adjusting Public Health and Social Measures in the Context of COVID-19: Interim Guidance. https://iris.who.int/bitstream/handle/10665/331773/WHO-2019-nCoV-Adjusting_PH_measures-2020.1-eng.pdf.

[2] Antunes R., Frontini R. (2021). Physical Activity and Mental Health in Covid-19 Times: An Editorial. Sleep. Med..

[3] Stockwell S., Trott M., Tully M., Shin J., Barnett Y., Butler L., McDermott D., Schuch F., Smith L. (2021). Changes in Physical Activity and Sedentary Behaviours from before to during the COVID-19 Pandemic Lockdown: A Systematic Review. BMJ Open Sport Exerc. Med..

[4] Smith L., Jacob L., Yakkundi A., McDermott D., Armstrong N.C., Barnett Y., López-Sánchez G.F., Martin S., Butler L., Tully M.A. (2020). Correlates of Symptoms of Anxiety and Depression and Mental Wellbeing Associated with COVID-19: A Cross-Sectional Study of UK-Based Respondents. Psychiatry Res..

[5] Gómez-Salgado J., Andrés-Villas M., Domínguez-Salas S., Díaz-Milanés D., Ruiz-Frutos C. (2020). Related Health Factors of Psychological Distress during the COVID-19 Pandemic in Spain. Int. J. Environ. Res. Public Health.

[6] Troyer E.A., Kohn J.N., Hong S. (2020). Are We Facing a Crashing Wave of Neuropsychiatric Sequelae of COVID-19? Neuropsychiatric Symptoms and Potential Immunologic Mechanisms. Brain Behav. Immun..

[7] Mukhtar S. (2020). Psychological Health during the Coronavirus Disease 2019 Pandemic Outbreak. Int. J. Soc. Psychiatry.

[8] Bull F.C., Al-Ansari S.S., Biddle S., Borodulin K., Buman M.P., Cardon G., Carty C., Chaput J.-P., Chastin S., Chou R. (2020). World Health Organization 2020 Guidelines on Physical Activity and Sedentary Behaviour. Br. J. Sports Med..

[9] Hamilton M.T., Healy G.N., Dunstan D.W., Zderic T.W., Owen N. (2008). Too Little Exercise and Too Much Sitting: Inactivity Physiology and the Need for New Recommendations on Sedentary Behavior. Curr. Cardiovasc. Risk Rep..

[10] Owen N., Sparling P.B., Healy G.N., Dunstan D.W., Matthews C.E. (2010). Sedentary Behavior: Emerging Evidence for a New Health Risk. Mayo Clin. Proc..

[11] Chekroud S.R., Gueorguieva R., Zheutlin A.B., Paulus M., Krumholz H.M., Krystal J.H., Chekroud A.M. (2018). Association between Physical Exercise and Mental Health in 1· 2 Million Individuals in the USA between 2011 and 2015: A Cross-Sectional Study. Lancet Psychiatry.

[12] Dunton G.F., Wang S.D., Do B., Courtney J. (2020). Early Effects of the COVID-19 Pandemic on Physical Activity Locations and Behaviors in Adults Living in the United States. Prev. Med. Rep..

[13] Ammar A., Brach M., Trabelsi K., Chtourou H., Boukhris O., Masmoudi L., Bouaziz B., Bentlage E., How D., Ahmed M. (2020). Effects of COVID-19 Home Confinement on Eating Behaviour and Physical Activity: Results of the ECLB-COVID19 International Online Survey. Nutrients.

[14] Constandt B., Thibaut E., De Bosscher V., Scheerder J., Ricour M., Willem A. (2020). Exercising in Times of Lockdown: An Analysis of the Impact of COVID-19 on Levels and Patterns of Exercise among Adults in Belgium. Int. J. Environ. Res. Public Health.

[15] Huckins J.F., da Silva A.W., Wang W., Hedlund E., Rogers C., Nepal S.K., Wu J., Obuchi M., Murphy E.I., Meyer M.L. (2020). Mental Health and Behavior of College Students During the Early Phases of the COVID-19 Pandemic: Longitudinal Smartphone and Ecological Momentary Assessment Study. J. Med. Internet Res..

[16] Zhang Y., Zhang H., Ma X., Di Q. (2020). Mental Health Problems during the COVID-19 Pandemics and the Mitigation Effects of Exercise: A Longitudinal Study of College Students in China. Int. J. Environ. Res. Public Health.

[17] Gallè F., Sabella E.A., Da Molin G., De Giglio O., Caggiano G., Di Onofrio V., Ferracuti S., Montagna M.T., Liguori G., Orsi G.B. (2020). Understanding Knowledge and Behaviors Related to COVID–19 Epidemic in Italian Undergraduate Students: The EPICO Study. Int. J. Environ. Res. Public Health.

[18] López-Bueno R., Calatayud J., Casaña J., Casajús J.A., Smith L., Tully M.A., Andersen L.L., López-Sánchez G.F. (2020). COVID-19 Confinement and Health Risk Behaviors in Spain. Front. Psychol..

[19] Pais-Ribeiro J.L., Honrado A., Leal I. (2004). Contribuição Para o Estudo Da Adaptação Portuguesa Das Escalas de Ansiedade, Depressão e Stress (EADS) de 21 Itens de Lovibond e Lovibond. Psicol. Saúde Doenças.

[20] Lovibond P.F., Lovibond S.H. (1995). The Structure of Negative Emotional States: Comparison of the Depression Anxiety Stress Scales (DASS) with the Beck Depression and Anxiety Inventories. Behav. Res. Ther..

[21] Lovibond S.H., Lovibond P.F. (1995). Manual for the Depression Anxiety Stress Scales.

[22] Aires L., Pratt M., Lobelo F., Santos R.M., Santos M.P., Mota J. (2011). Associations of Cardiorespiratory Fitness in Children and Adolescents with Physical Activity, Active Commuting to School, and Screen Time. J. Phys. Act. Health.

[23] Eisenmann J.C., Bartee R.T., Wang M.Q. (2002). Physical Activity, TV Viewing, and Weight in US Youth: 1999 Youth Risk Behavior Survey. Obes. Res..

[24] Craig C.L., Marshall A.L., Sjöström M., Bauman A.E., Booth M.L., Ainsworth B.E., Pratt M., Ekelund U.L.F., Yngve A., Sallis J.F. (2003). International Physical Activity Questionnaire: 12-Country Reliability and Validity. Med. Sci. Sports Exerc..

[25] Campaniço H.M.P.G. (2016). Validade Simultânea Do Questionário Internacional de Actividade Física Através Da Medição Objetiva Por Actigrafia Proporcional. Master’s thesis.

[26] World Health Organization (2020). WHO Guidelines on Physical Activity and Sedentary Behaviour: At a Glance.

[27] Buysse D.J., Reynolds III C.F., Monk T.H., Berman S.R., Kupfer D.J. (1989). The Pittsburgh Sleep Quality Index: A New Instrument for Psychiatric Practice and Research. Psychiatry Res..

[28] João K.A.D.R., Becker N.B., de Neves Jesus S., Martins R.I.S. (2017). Validation of the Portuguese Version of the Pittsburgh Sleep Quality Index (PSQI-PT). Psychiatry Res..

[29] Gomes A.A., Marques D.R., Meiavia A.M., Cunha F., Clemente V. (2018). Psychometric Properties and Accuracy of the European Portuguese Version of the Pittsburgh Sleep Quality Index in Clinical and Non-Clinical Samples. Sleep Biol. Rhythm..

[30] Lee M.-S., Shin J.-S., Lee J., Lee Y.J., Kim M., Park K.B., Shin D., Cho J.-H., Ha I.-H. (2015). The Association between Mental Health, Chronic Disease and Sleep Duration in Koreans: A Cross-Sectional Study. BMC Public Health.

[31] García-García J., Mañas A., González-Gross M., Espin A., Ara I., Ruiz J.R., Ortega F.B., Casajús J.A., Rodriguez-Larrad A., Irazusta J. (2023). Physical Activity, Sleep, and Mental Health during the COVID-19 Pandemic: A One-Year Longitudinal Study of Spanish University Students. Heliyon.

[32] Rees-Punia E., Newton C.C., Rittase M.H., Hodge R.A., Nielsen J., Cunningham S., Teras L.R., Patel A. (2021). Prospective Changes in Physical Activity, Sedentary Time and Sleep during the COVID-19 Pandemic in a US-Based Cohort Study. BMJ Open.

[33] Vogel E.A., Zhang J.S., Peng K., Heaney C.A., Lu Y., Lounsbury D., Hsing A.W., Prochaska J.J. (2022). Physical Activity and Stress Management during COVID-19: A Longitudinal Survey Study. Psychol. Health.

[34] Martínez-de-Quel Ó., Suárez-Iglesias D., López-Flores M., Pérez C.A. (2021). Physical Activity, Dietary Habits and Sleep Quality before and during COVID-19 Lockdown: A Longitudinal Study. Appetite.

[35] Bener A., Morgul E., Tokaç M., Ventriglio A., Jordan T.R. (2024). Sleep Quality, Quality of Life, Fatigue, and Mental Health in COVID-19 Post-Pandemic Türkiye: A Cross-Sectional Study. Front. Public Health.

[36] Shillington K.J., Vanderloo L.M., Burke S.M., Ng V., Tucker P., Irwin J.D. (2024). Factors That Contributed to Ontario Adults’ Mental Health during the First 16 Months of the COVID-19 Pandemic: A Decision Tree Analysis. PeerJ.

[37] da Estrela C., McGrath J., Booij L., Gouin J.-P. (2021). Heart Rate Variability, Sleep Quality, and Depression in the Context of Chronic Stress. Ann. Behav. Med..

[38] Vestergaard C.L., Skogen J.C., Hysing M., Harvey A.G., Vedaa Ø., Sivertsen B. (2024). Sleep Duration and Mental Health in Young Adults. Sleep Med..

[39] Zagaria A., Serena S., Musetti A., Rapelli G., De Gennaro L., Plazzi G., Franceschini C. (2024). Poor Sleep Hygiene Practices Are Associated with a Higher Increase in Sleep Problems during the COVID-19 Pandemic: A Latent Change Score Model. J. Sleep Res..

[40] Paolucci E.M., Loukov D., Bowdish D.M.E., Heisz J.J. (2018). Exercise Reduces Depression and Inflammation but Intensity Matters. Biol. Psychol..

[41] SFM C., Van Cauwenberg J., Maenhout L., Cardon G., Lambert E.V., Van Dyck D. (2020). Inequality in Physical Activity, Global Trends by Income Inequality and Gender in Adults. Int. J. Behav. Nutr. Phys. Act..

